# Arthritis-Related Support in a Social Media Group for Quilting Hobbyists: Qualitative Study

**DOI:** 10.2196/11026

**Published:** 2018-10-03

**Authors:** Norina Gasteiger, Rebecca Grainger, Karen Day

**Affiliations:** 1 Health Systems School of Population Health The University of Auckland Auckland New Zealand; 2 Department of Medicine University of Otago Wellington New Zealand

**Keywords:** arthritis, social media, leisure, hobbies, peer group

## Abstract

**Background:**

People with arthritis are increasingly seeking support online, particularly for information about social role participation while experiencing symptoms of chronic arthritis. Social media enables peer-to-peer support on how serious leisure (eg, hobbies such as quilting) can be adapted to allow participation. Research is needed to understand what type of peer support is provided online and how this support occurs.

**Objective:**

The aim of our study was to explore what kind of support is offered by fellow hobbyists (with or without arthritis) in response to requests for advice in a social media group.

**Methods:**

Three vignettes were posted on a Facebook quilting group regarding arthritis-related symptoms or impairments that affect how people quilt. A Facebook Insights report was used to examine the groups’ demographics. Responses to the vignettes were thematically analyzed.

**Results:**

The members of the quilting Facebook group were mostly women (18,376/18,478, 99.45%), aged 55 to 64 years, and most were located in the United States. In response to the vignettes, the 22 participants predominantly offered emotional support and shared information. Participants shared their real-life experiences and creative means in adapting medical advice to their crafting. More than half (30/54, 56%) of the advice that was offered aligned with the OrthoInfo medical best practice guidelines relevant to the vignettes.

**Conclusions:**

Serious leisure social media groups can be useful forums for sharing information about arthritis-related issues. People do respond to requests for support and information, although there is a difference between quilting support (eg, “I need a new iron, what should I buy?”) and health support (eg, “I have arthritis, what scissors should I buy?”). People provide emotional support for life events on serious leisure social media platforms (eg, offering condolences when a person states that she is making a memory quilt), and this extends to health issues when group members reveal them.

## Introduction

### Background

Quilters take their craft very seriously. Serious leisure is defined by Stebbins [1] as a “systematic pursuit of an amateur, hobbyist, or volunteer activity sufficiently substantial, interesting, and fulfilling for the participant to find a (leisure) career there acquiring and expressing a combination of its special skills, knowledge, and experience.” Quilting is more than a sewing craft. It is the coming together of like-minded crafters to build a community of people who reflect culture, remember history, create art, and build quilted products demonstrating a range of skills from novice to expert. Quilters are hobbyists who find benefit and pleasure in what they do and are committed to the development of knowledge, skills, and experience associated to enable participation in their hobby either at a serious or casual level [2]. Similarly, people living with long-term health issues acquire extensive knowledge about their condition, solve problems, and become an expert at handling their health [3].

Arthritis and painful musculoskeletal conditions are common long-term health issues that are a leading cause of disability worldwide [4,5]. Inflammatory arthritis (such as rheumatoid arthritis and the spondyloarthropathies) and osteoarthritis are leading causes of pain and can impair participation [6]. Arthritis often occurs in a person’s most productive age range in adulthood and affects function and participation in everyday activities and hobbies [7,8]. Evidence-based medicine has resulted in diagnostic and treatment guidelines to enable knowledge translation from research to practice to improve health outcomes. From a clinician’s perspective, adherence to evidence-based medicine is fraught [9,10] with adherence issues such as unpleasant side effects of medications. Medication adherence for rheumatoid arthritis, for example, can be as low as 30%, and interventions have been designed to improve adherence [11]. Translation of guidelines and medical advice into practice by patients encounters difficulties due to inequities (education and socioeconomic), low health literacy, and compromised cognitive abilities due to the illness itself and side effects of the medication [9].

Social media provides opportunities for people with health issues to elicit and share support and advice to adapt their activities to accommodate their condition. People use a variety of platforms (eg, Facebook, Twitter, and YouTube) to find and exchange information about health issues, elicit support, and improve self-management of their health issues [12]. People also join disease-specific groups provided by clinicians or patients to learn about their health issues and elicit and/or provide support [13]. Many join communities such as PatientsLikeMe, where individuals with similar conditions can share, view, and discuss their data and health issues, providing peer-to-peer support [14]. The literature does not explore serious leisure platforms (eg, quilting groups) where people may share and exchange information and support about long-term conditions as part of their participation in their leisure activity.

### Objective

Our research question was as follows: What kind of support is offered by fellow hobbyists (with or without arthritis) in response to requests for advice in a social media group?

## Methods

### Study Setting

A quilting Facebook group was chosen as the research site because older women are predominantly affected by arthritis. Quilters are often older women and increasingly use Facebook groups for quilting advice [15]. The Quilting in America 2017 survey estimates there are 7 to 10 million quilters in the United States, most are female, have a mean age of 63 years, and are educated (70% attended college or university) and affluent [15]. Compared with the 2014 survey, time spent online in quilting-related sites has increased from 2.5 to 7.9 hours weekly and Facebook users from 14% to 50% [15]. Textile art has been shown to enhance well-being and quality of life and can have therapeutic benefits [16,17]. Given the widespread engagement with quilting-related social media sites and possible health benefits, a quilting Facebook group is appropriate for this research.

### Recruiting Participants

To identify a group for our study, we reviewed 100 quilting groups on Facebook, resulting from a search using “quilt” as a keyword. A total of 10 groups were false positive results (eg, blanket knitting) and were excluded. Groups with 15,000 members or less were excluded from consideration for our study, as we were looking for a large enough group to elicit conversations with a likelihood of at least 10 responses to each of our scenarios (as per Table 1).

A general quilting group was deemed most appropriate because of the broad mix of skills (that was not evident in many of the specialty groups, eg, rag quilting) and range of experience (from beginner to expert). This left 6 qualifying groups to approach. The groups were approached in order of membership size. The administrators of the first 2 groups considered the researchers’ invitation but declined to participate. It was decided that a smaller group among the specialty groups might be more flexible. Group sizes ranged from 563 to 150,000 members. A relatively small group of 18,478 members, dynamic in its conversation (>10 posts per day), with a growing membership, and that had previously posted arthritis-related questions, was deemed appropriate. It was a closed group, did not allow selling or self-promotion, and its function was primarily as a quilting support group. The nature of the specialty was broad, leveraging general quilting skills, for example, piecing and long-arm or free form quilting plus specialty skills.

The main administrator was approached by a private message and, after consultation with her group members, granted permission for the research. The administrator posted a comment indicating that KD had approached her to conduct research in the group saying, “Before allowing this in the group, I wanted to check with all of you. Please comment on this post and tell me if you’re OK with this or not.” A total of 78 people responded to it. Most group members were supportive in their comments (61), and 28 people clicked the *like* button for the initial post (we did not match these *likes* with the comments). A total of 4 posters indicated that they had arthritis-related health issues that affected their ability to quilt. They later commented in response to the vignettes. Most of the *likes* in the discussion were by KD and the group administrator to acknowledge comments. Some gave reasons for their support (they have arthritis, could learn something, and were happy to help). After 19 posts, new posters started indicating *no* with comments about medical research not being appropriate for a quilting group, and others wanted to avoid discussion about their own health issues when doing their craft. Thereafter, the conversation became mixed.

**Table 1:** Types of quilting groups reviewed for recruitment.

Group type	Large groups^a^ (N=89), n (%)	Small groups^b^ (N=64), n (%)
Buying and selling	12 (14)	9 (14)
Beginners	4 (5)	2 (3)
On the basis of a specific person’s work	6 (7)	4 (6)
Specialty	45 (51)	36 (56)
General quilting	22 (25)	13 (20)^c^

^a^Greater than 15,000 members.

^b^15,000 members or less.

^c^An additional 3 were limited to one country, and one was limited to a research project.

A total of 3 members conversed with those who said *no*, defending the research, for example, the members could scroll over what was not of interest. Moreover, 2 people reiterated the purpose of the group as a support group for quilting and that other topics were not to be discussed. Near the end of the conversation, 1 person suggested that the group administrator make her own decision and this was supported by 2 others. Others offered alternative solutions, for example, researcher sets up her own Facebook page, whereas others were concerned that if permission was granted, it would be a *slippery slope* or would *take away from sharing and quilting*. The administrator made her decision after 3 days and consented to the research.

The University of Auckland Human Participants Ethics Committee approved this study on September 12, 2017 (reference 019783). The administrator of the Facebook group signed a consent form on behalf of the group members. Group members were informed that they could participate in the research by responding to scenarios. An explanation was provided with each post informing members that responses were being collected for research. A link to a summary of the study and full participant information sheets was also included in each post [18]. One researcher was a member of the group (KD) and collected the data, which were sent to the other researchers for analysis and reflection (NG and RG). The discussion threads have since been deleted to ensure privacy of the participants, as quotes used in this study could lead to identification of research participants by new or existing members of the Facebook group.

### Data Collection

**Figure 1:**
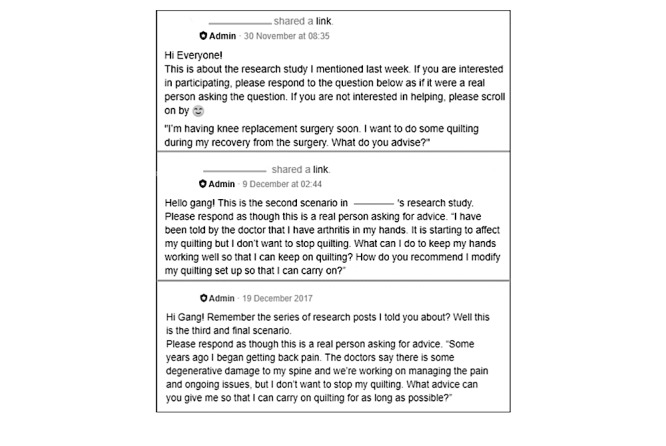
Screenshot of the vignettes as posted in the quilting group.

Overall, 3 vignettes (case scenarios) were posted as threads in the Facebook group, to which members responded (Figure 1).

The rules of the quilting group stated the following: no selling or self-promotion is allowed, no politics, respect copyright, and be kind and supportive. The researchers designed vignettes to respect the primary role of the group, that is, quilting sharing and support. Vignettes were appropriate, as they enable sensitive qualitative data collection and are an effective tool for eliciting judgments and perceptions [19]. The vignettes alluded to arthritis-related issues that affect how people quilt and included a person undergoing knee joint arthroplasty (replacement) for knee osteoarthritis, a person with hand pain from hand osteoarthritis, and a person with chronic nonspecific lower back pain (Figure 1).

The vignettes were developed by a nurse (KD) and rheumatologist (RG). After no responses to the first vignette, it was removed, shortened, and simplified (response instructions were replaced by a link to KD’s blog). The short version encouraged discussion. Vignettes were left on the Facebook page (November 30, 2017 and December 9 and 19, 2017) for approximately 1 week until no new responses were made. KD posted a summary of each discussion a week after the initial post to allow for further commentary or correction, which did not occur.

### Data Analysis

**Figure 2:**
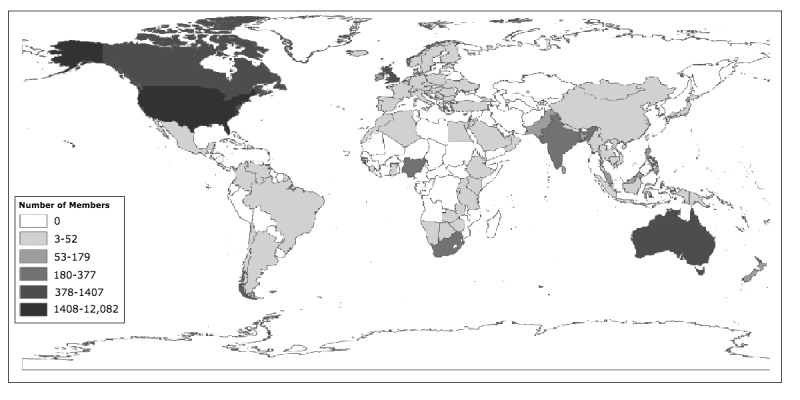
Location of quilting closed Facebook group members around the world.

Before the vignettes were posted, the administrator of the Facebook group extracted demographic data from the group using the *Page Insights* function. The resulting Excel spreadsheet was sent to the researchers for quantitative analysis of demographic characteristics, including summary statistics of age and gender. A map of the distribution of group members internationally (Figure 2) was created by NG using ArcGIS (Environmental Systems Research Institute, 2017, ArcGIS Desktop Help 10.5.1) [20].

Responses to each thread were screenshot, cut, and pasted and then transcribed onto an MS Word document for analysis and reflection using Williams’ reflective diary guide [21] (see Textbox 1) to conduct an inductive thematic analysis [22]. The discussion threads and reflections were analyzed thematically. This consisted of coding the qualitative data and identifying themes that maintain the richness of the data and accurately represent and explain the phenomena [23]. Interactions between themes were examined and their relevance to the research aim was explained. All 3 researchers reflected on the data and made comments on the analysis.

The discussion thread data were compared with medical best practice for each vignette using the following steps:

- The peer advice from the posted comments was coded (eg, exercise or medication use) and grouped by codes in a table.
- Information for patients about recommended management and self-care from OrthoInfo [24] was summarized by RG (a rheumatologist) in MS Word. OrthoInfo is a website for lay people, developed and reviewed by members of the American Academy of Orthopaedic Surgeons (AAOS) and provides evidence-based information about treatment of, and self-care for, musculoskeletal conditions [24]. OrthoInfo was chosen because the AAOS is the world’s largest professional association for musculoskeletal specialists, and the information has undergone peer review by an expert panel of 13-member editorial board [25].
- The self-care management options (ie, recommendations that could be implemented by an individual) proposed by OrthoInfo were identified from the summary.
- Coded peer advice that was congruent with self-care recommendations from OrthoInfo were matched in Table 2.
- Summary statistics about frequency of peer advice congruent (or not) with OrthoInfo recommendations were prepared.

**Reflection tool questions.**

At the end of the period covered by each vignette, note down and reflect on:

- The most interesting issue
- What worked well (eg, significant achievements)
- What did not work well
- The most puzzling or confusing issue
- The most unexpected issue
- Any risks and threats to the project
- Any opportunities for the project
- Any implications for the principles and purpose underpinning the project
- Differences between the plan and the action
- What was noticed but not addressed? Why? Would addressing that have improved the outcome?
- Any other comments or observations

**Table 2:** Comments and replies for each vignette.

Vignettes	Participants (n=22), n (%)	Comments (n=27), n (%)	Replies (n=11), n (%)
1	8 (36)	8 (30)	4 (36)
2	5 (23)	8 (30)	5 (46)
3	11 (50)	11 (40)	2 (18)

## Results

### The Context

Most quilting groups were international (of the 100 groups that we analyzed, only 3 were limited to 1 country). The Facebook group had 18,478 members when the first vignette was posted. Facebook Page Insights showed that the country with the most members was the United States of America, followed by Canada, the United Kingdom, and Australia. Members were spread all over the world, with high numbers in India, China, Europe, South Africa, and Nigeria and lowest density of members in South America, China, Europe, and parts of Africa (Figure 2). Thus, the group consisted of members from different countries (developed and developing) although membership was predominantly from the United States.

**Figure 3:**
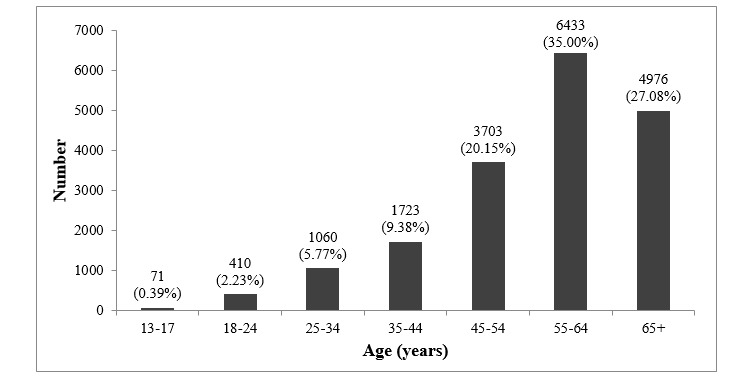
Number of female members by age in quilting closed Facebook group.

**Figure 4:**
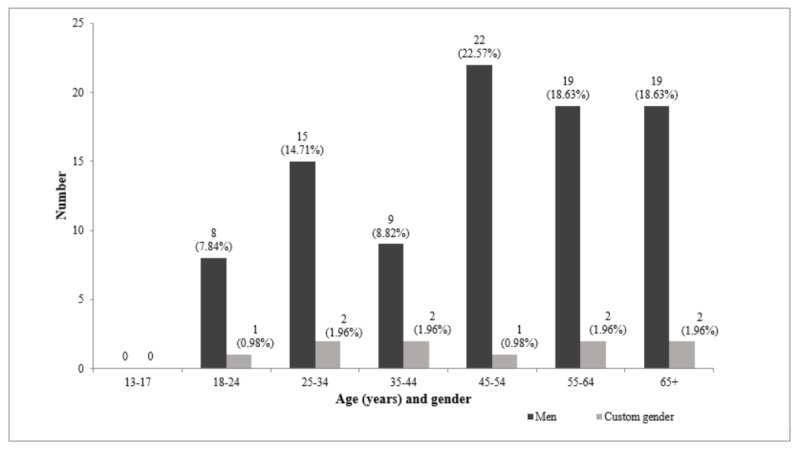
Number of male and custom (gender diverse) members by age in quilting closed Facebook group.

Almost all of the members from the quilting Facebook group identified as females (18,376/18,478, 99.44%; Figure 3), with a very small proportion identifying as males (92/18,478, 0.49%) or custom (10/18,478, 0.05%; Figure 4). The biggest proportion of female members (6433/18,478, 34.81%) was aged 55 to 64 years and for male members (21.6%) was 45 to 54 years. Those with custom genders were distributed more evenly across the age categories. None of the members who identified as male or custom were in the youngest age group, that is, 13 to 17 years.

Of the top 100 contributors to the quilting group, the posts ranged from 0 to 10 (87 members), 11 to 20 (6 members), and more than 20 (3 members). The top commenters (responses to posts) ranged from 13 to 99 (69 members), 100 to 199 (17), 200 to 299 (7), and 300 to 721 (4). Furthermore, 2 of the respondents to our vignettes were among the top 100 contributors. While 1 person posted in vignette 1 (V1) and vignette 2 (V2), had 6 posts, and 518 comments before the research started, the other person posted in vignette 3 (V3), had no posts, and 270 comments before the research started. We were unable to reliably collect demographic data from the Facebook pages of individual participants because of most privacy settings blocking access to this kind of information. The Facebook Insights report did not supply demographics.

### Discussions Relating to the Vignettes

A total of 22 people participated in the study by commenting or replying to comments in the vignettes (Table 1). There were 38 contributions (comments and replies) in total across the vignette discussions, with 71% (27/38) original posts and 29% (11/38) comments or responses. Participation was similar in all 3 case scenarios (V1, V2, and V3, n=14 each). Participants contributed an average of 1.7 times each. Each vignette was only posted once.

For 14 responses of V1, 2 participants responded to a comment by another participant or the researcher. There were 1 to 3 likes per comment—most of them were from the researcher and the group administrator to acknowledge the comments and encourage more comments. KD asked 8 questions in response to posts and received 1 reply. One participant also contributed to the consent conversation and was one of the top 100 contributors to the group. Of the 13 comments for V2, 1 participant posted 3 comments and responded to the researcher’s question with 3 more comments. She also responded twice to comments by others.

The *likes* were mostly given by the administrator but 2 were given by 1 of the participants plus someone who did not post a comment. Again, 1 participant also contributed to the consultation conversation and was 1 of the top 100 group contributors. Of 13 comments of V3, 1 was a question by the researcher to stimulate discussion (for which there was 1 reply by someone who had already posted) and 4 were responses to comments. No one posted more than once. The *likes* as for the other vignettes were given by the researcher and the administrator to encourage more participation. KD responded to 2 comments by asking questions, but there were no replies. In addition, 2 participants had contributed to the consultation conversation and were also in the top 100 contributors. A total of 2 participants responded to both vignettes 1 and 2. Of those who participated in the consultation conversation, 2 responded to V1, and 1 responded to V3. No video links were shared. In addition, 2 website links were provided—1 in V2 for examples of spring-loaded scissors and 1 in V3 to a posture guide for computer use.

### Emerging Themes

#### Emotional Support

Members offered emotional support in V1 and V2 through encouragement and reassurance. Members responding in V1 made encouraging comments such as wishing the hypothetical person good luck for their knee replacement surgery. Some members were encouraging about undergoing therapy by emphasizing that this was crucial to good recovery. Another member justified that she gave advice to encourage a person to continue quilting. She later reinforced the value of offering support:

I hope this advice encourages your “pretend” person to continue quilting...My pleasure if it helps someone else.

Reassuring comments in V1 supported the decision for having knee replacement surgery and emphasized that recovery is only temporary and short term. Some members referenced activities that they were able to do again within a short time frame post surgery:

Was back at sewing machine for short periods 3 weeks post op.

Comments that encouraged positive mindset were made in response to V1. These related to participants staying positive and hopeful throughout recovery and reassured that they were content during this time:

Keep cheerful, and remember you’ll soon be able to continue normal activities...look forward to quilt shows for more ideas for even more quilts to make.

#### Information Sharing

Participants shared a range of information on arthritis-related topics. Advice offered in V1 was specific to recovery, whereas that given in V2 and V3 was related to everyday life with arthritis. This included medical information, adapting quilting to their condition, and using assistive tools. Experience was mentioned to support a recommendation or to acknowledge a lack of knowledge. Those who had lived experience provided more in-depth and personal information and provided external links to information as well.

Medical information across the cases included additional treatments such as massage, exercises such as yoga or Pilates, continuing with quilting, dance, correct posture, pain and symptom management through the use of arthritis gloves, anti-inflammatory gel, heated massage pillows, and resting. Some recommendations, such as this advice from V3, were couched in humor:

Get up and boogie. LoL. Or limbering yoga moves.

Members commenting in V1 emphasized the importance of trusting medical professionals and following instructions. They specifically recommended following medical instructions, asking questions, and attending physiotherapy appointments:

I fully followed all medical instructions, and had asked enough questions to know what to expect.

Members commenting in V1 also valued preparation, as they assumed that exercise would be detrimental following a knee replacement surgery. They suggested resting, recovering, and staying off the stairs. As some of their craft rooms were downstairs, they recommended being prepared with kits, handwork, and smaller quilts to work on. They also suggested having someone retrieve their gear when necessary:

If I needed something from my craft area I got someone else to fetch it for me until I was allowed on the stairs again. I suppose the best advice I could give would be to resolve yourself to limited activity for a short period of time and plan toward that end.

Members’ comments in all 3 cases emphasized the importance of taking breaks. While comments in V1 referred to prioritizing recovery, those in V2 related to resting during flare-ups until they passed and taking breaks to stretch hands. Comments by members in V3 acknowledged that they were unable to quilt on some days due to back pain and would watch people quilt instead:

Choose your battles. Some days I can deal with it and some days I can’t. When I can’t, I watch other people sew on YouTube :D.

Responses to V3 also revealed the importance of posture to minimize back pain. Interestingly, members suggested applying good office ergonomics to their quilting environments. This included correct posture by using back braces or pillows to sit straight and adjusting their chairs. In addition, 1 member suggested tilting the sewing machine forward to alleviate stress on the back, shoulders, and neck. Another transferred posture advice from their employer to their sewing:

I worked at a fortune 500 company and posture is critical working at a station all day working on a computer. Along w/your breaks, make sure you have good posture at your sewing station. Sit up straight and make sure your chair adjusts for good posture.

The use of assistive and labor-saving tools were recommended in V2, as hands often felt weak, tired, or sore. Participants suggested using branded spring-loaded scissors, ergonomic rotary cutters, and suction cups to hold down rulers. Members provided an external link to purchase the spring-loaded scissors:

Absolutely get spring-loaded scissors. I also use the smaller spring-loaded nippers for handwork/quilting-so much easier than regular scissors. The blades on the nippers are so pointy that I can use them to rip stitches out which is much easier on my hands.

Experience with arthritis or lack thereof was referred to, either to reinforce or endorse information or to display a lack of certainty about advice. For V1, 4 of the 9 people participating in the conversation indicated they had had knee replacement surgery done. Of the 5 people conversing in V2, 1 stated that she had rheumatoid arthritis and another referred to her experience without saying if she had arthritis. Of the 12 people who responded to V3, 7 referred to their own experience but did not indicate if they have arthritis. Half of the participants either had arthritis or spoke from an experience that informed their responses. Members with lived experience also endorsed others’ advice:

I had knee replacement 1 year ago yesterday. I agree with the ladies above me.

**Table 3:** Alignment between best practice and advice given.

Vignette and best practice				Times mentioned, n (%)
**Vignette 1**				
	**Knee replacement (n=22)**			
		Modify home or environment	9 (41)	
		Wound care and balanced diet	0 (0)	
		Resume normal activities	1 (5)	
		Exercise program	5 (23)	
		Use gait aid until advised to discontinue, graduated walking program	0 (0)	
		Other advice	7 (32)	
**Vignette 2**				
	**Hand arthritis (n=9)**			
		Medication	1 (11)	
		Splinting	1 (11)	
		Other advice	7 (78)	
**Vignette 3**				
	**Back pain (n=23** **)**			
		Physical therapy	3 (13)	
		Braces	2 (9)	
		Yoga or Pilates	3 (13)	
		Aerobic exercise	1 (4)	
		Healthy weight, avoid smoking, chiropractic, and care with lifting	0 (0)	
		Good posture	4 (17)	
		Other advice	10 (44)	

Conversely, some people without arthritis or those who had not tried a treatment or tool acknowledged their lack of experience. For example, 1 member suggested trying arthritis gloves but then acknowledged that she has no lived experience with them:

I’m not much help.

#### Medical Guideline Alignment

Of all of the self-care advice offered, just over half (30/54, 56%) aligned with the OrthoInfo recommendations for knee replacement surgery (V1: 15/22, 68%), hand arthritis (V2: 2/9, 22%), and back pain (V3: 13/23, 57%; see Table 3).

In several instances, participants appeared to offer advice on adapting both the environment and the quilt making in ways that aligned with the OrthoInfo medical guideline. Most of the information that did not align was valuable but was too diverse and quilting-specific. This was particularly evident in V1, where 4 comments referred to resting after a knee replacement surgery whereas the guideline suggests resuming normal activity. Moreover, 2 other comments included advice on pain management, which was not found in the OrthoInfo guideline.

With regard to quilting-specific advice, the guideline did not mention assistive tools for hand arthritis, yet in V2, most of the comments consisted of suggestions for adapting their quilting environment through the use of tools such as suction cups to hold rulers down or spring-loaded scissors. Other comments included resting and keeping fingers active.

Comments that did not align in V3 were diverse. These included varying positions, wearing supportive shoes, finding a comfortable chair or one that is on wheels, taking breaks, and using an orthopedic pillow. A lack of commentary on the best practice recommendation of *care with lifting* in V3 may have been explained by members having craft rooms dedicated to their quilting or assuming that others have craft rooms. These bespoke craft rooms may alleviate the need to carry heavy equipment.

## Discussion

### Principal Findings and Comparison With Prior Work

#### Participation Pattern

Low participation rates were noted, as only 22 people or 0.12% (22/18,478) of the Facebook group members participated in the study. A combination of factors may explain this. There was a low rate of posting for the top 100 contributors (from 0-29 posts) and commenting (4 people had made more than 300 comments). These low posting and commenting rates were reflected in the vignette conversations, where 4 of the top 100 contributors responded to the vignettes. As indicated in the conversation of the consent consultation, some members did not see the relevance of the research to their quilting, and others felt that their quilting group was not the right place for research or medical discussions. Some people used their quilting and the group to avoid life issues while pursuing their hobby. There were more than 10 posts a day in this group, with top recent posts receiving 18 to 61 comments. It is possible that the vignettes were missed, as they were not pinned (prioritized) posts. As the frequency of posting and commenting was already low in the group, it is not surprising that only 22 people responded to the vignettes.

Could the vignettes themselves have contributed to low participation? They were constructed to mimic requests for assistance and advice in other Facebook groups that KD belongs to, including other quilting groups. Posts were pithy and extended a clear question for advice, assistance, or support. Ethics about social media research influenced the decision to use a hypothetical person requesting advice. Informed consent and transparency about data gathering are a prerequisite for research [26]. Consequently, the researcher who was a group member was positioned not only as a quilter but also as a researcher. Trust was important as exhibited by the response of the first group that was approached, and concern during the consent consultation that allowing research in the group would be the start of a slippery slope. To achieve informed consent, it was necessary to provide a link to the website (leading to participant information and consent statements) as part of the vignettes that were posted, resulting in an implied emphasis on the *pretend* person posting the vignette questions.

People using social media enjoy a degree of invisibility and anonymity, especially in closed discussion forums such as closed groups on Facebook. This is particularly valuable to people with long-term health issues who do not need to disclose much about themselves to gain support or find information [27]. They are able to seek information and emotional support, form relationships, affect behavior appropriate to the profile they are projecting, for example, as a quilter, and tell their story on their own terms. Social media becomes a level playing field for people with disabilities or health issues that affect their ability to pursue a hobby. It is possible that participants living with arthritis did not want to reveal their health issues to protect their quilting profile.

#### Emotional Support

Participants offered emotional support through reassuring and encouraging comments. Some participants justified their contributions by acknowledging that their encouragement is valuable and could ultimately benefit others. Receiving support (online or offline) has been linked to improved health outcomes [13]. The act of offering others support online can act as a vehicle for emotional support, for example, self-worth is enhanced by helping others [28]. Members indicated that recovery after knee replacement surgery is short term and encouraged a positive mindset. This exemplifies a form of advice that Mendelson [29] identified regarding continuity of life and events (despite diagnosis). Emotional support and, more specifically, advice on continuity of life are beneficial, as anxiety and depression are prevalent among people with rheumatoid arthritis [30]. It appears that acts of providing and receiving emotional support are valued by group members who responded to our vignettes.

#### Information Sharing and Medical Guideline Alignment

Is best practice medical advice accurately shared by quilters living with arthritis? About half of the advice offered aligned with medical best practice for self-care. Noncompliance for physiotherapy and physical exercise programs are common and may be as high as 70% [31,32]. People who assume exercising would not help or experienced barriers to exercising, for example, pain, were less compliant [31,32]. Some participants who responded to the surgical vignette indicated that although they had prepared themselves for convalescence, the blur between postsurgical pain and the need to rest may explain early avoidance of walking on stairs. As our research did not set out to measure offline behavioral changes or improvements in health outcomes, we are not able to draw conclusions about any effects of information sharing.

From a medical perspective, information shared online can be inaccurate, inappropriate, and misinformed [28,33,34]. The nonspecificity of the comments by our participants meant that they were open to interpretation by other members and did not consider potential safety impacts. For example, recommendations to tilt the sewing machine may have been creative or helpful, but there was no conversation about the possibility of the machine falling onto the user’s lap or the development of an ergonomically tilted machine by a manufacturer. People have the opportunity to become more informed and empowered with access to health-related information online, but they also are at risk from the consequences of incorrect information because of the volume of shared advice or an inability to discern good from bad advice [35]. With an increase of some age groups using social media for health-related information [36,37], health professionals should be prepared to assist their patients in discerning the quality of information available online [35,38].

Some of the advice that did not align and carried minimal safety risk was still considered to be valuable. This was exemplified in the suggestion for using assistive tools, for example, spring-loaded scissors or suction pads on rulers, which were not explicitly included in the OrthoInfo guideline [24]. Best practice guidelines differ, as evidenced in other guidelines recommending the use of joint protection techniques and assistive tools for the nonpharmaceutical management of hand osteoarthritis [39]. Although some advice with minimal safety risk is useful, it is important to recognize that the advice offered online might raise safety concerns, as advice is open to interpretation and implementation by those who read it. Clinicians cannot be present in every social media platform, and concern for accuracy of information sharing can be mitigated by teaching clinicians about the value of serious leisure for well-being and how to help their patients to critique available support.

### Limitations

This was an exploratory study. We acknowledge that there is a plethora of health-related social media groups and services. We were interested in how people integrate their health-related information and support-seeking into their serious leisure activities, using quilting as context. The sample size was small, but the findings revealed that people do share health-related information and customize it to their serious leisure hobby such as quilting, for example, computer ergonomics applied to posture at a sewing machine to avoid back ache. The findings can be transferred to similar settings, for example, other social media platforms such as Twitter and should be further investigated on a larger scale and in more depth, at which point generalizability of findings and clearer representation can be explored.

As an exploratory study, we used only 1 Facebook group and chose not to compare, for example, Facebook with Twitter, Instagram, Pinterest, or Reddit. Now that it has been suggested that people who participate in serious leisure in the form of quilting do fairly and accurately share health-related information, further studies should be completed using different platforms, comparing different groups in the same platform, and comparing closed and public groups on Facebook.

Participants responded to hypothetical scenarios. KD had observed health-related (and indeed, arthritis-related) conversations in quilting groups on Facebook and crafted the scenarios to closely resemble authentic questions. The scenarios were overtly from a researcher to avoid concerns about deception, as group members are militant about remaining on topic, that is, talking only about quilting-related topics. The obvious presence of the researcher may have resulted in self-selection bias.

Bias could have been introduced by self-selection for participation. Editing of posts and comments (to come across as more helpful, polite, and informed, or to present a profile that pleased the researcher and other quilting group members) could have introduced bias. The anonymity or crafting of a profile that is more positive than in real life, for example, not revealing health issues may have resulted in some people not participating in the research because they did not want to reveal their health issues. As our analysis of 100 Facebook quilting groups only revealed open pages that were linked to individual quilters (ie, not to actual groups or sets of followers that one might be more likely to find on Twitter or Instagram), it is possible that this research cannot be repeated outside a formal group if repeated on Facebook.

### Conclusions

In our exploratory study, we set out to find out what information people share about specific health issues that affect a serious leisure activity such as quilting and how they elicit and provide support to one another to continue with their quilting. People do respond to requests for support and information, although there is a difference between quilting support (eg, I need a new iron, what should I buy?) and health support (eg, I have arthritis, what scissors should I buy?). People provide emotional support for life events in serious leisure social media platforms (eg, offering condolences when a person states that she is making a memory quilt), and this extends to health issues when group members reveal them.

Future research can be informed by our research. The next step is to create a survey that incorporates theories of peer-to-peer support, serious leisure and serious leisure flow, empowerment, and evidence-based health care to establish the utility of online serious leisure groups for supporting a health issue. Future research could explore the extent to which support provided online has the ability to adapt offline behaviors.

## Abbreviations

AAOS: American Academy of Orthopaedic Surgeons
V1: vignette 1
V2: vignette 2
V3: vignette 3
